# Goniometry and Limb Girth in Miniature Dachshunds

**DOI:** 10.1155/2016/5846052

**Published:** 2016-06-15

**Authors:** Stephanie A. Thomovsky, Annie V. Chen, Alecia M. Kiszonas, Lori A. Lutskas

**Affiliations:** ^1^Department of Veterinary Clinical Sciences, Washington State University School of Veterinary Medicine, Ott Road, Pullman, WA 99164, USA; ^2^USDA-ARS, Washington State University School of Veterinary Medicine, Ott Road, Pullman, WA 99164, USA

## Abstract

*Purpose.* To report the mean and median pelvic limb joint angles and girth measurements in miniature Dachshunds presenting with varying degrees of pelvic limb weakness secondary to thoracolumbar intervertebral disc extrusion.* Methods.* 15 miniature Dachshunds who presented to WSU-VTH for thoracolumbar disc extrusion. Dachshunds varied in neurologic status from ambulatory paraparetic to paraplegic at the time of measurements.* Results. *There were no significant differences in joint angles or girth among the three groups (ambulatory paraparetic, nonambulatory paraparetic, or paraplegic) (*P* > 0.05). When group was disregarded and values for extension, flexion, and girth combined, no differences existed.* Conclusions.* Goniometry and limb girth measurements can successfully be made in the miniature Dachshund; however, the shape of the Dachshund leg makes obtaining these values challenging. There were no differences in joint angle or girth measurements between dogs with varying neurologic dysfunction at the time of measurement.

## 1. Introduction

Goniometry, the measurement of joint angles, has been used as a staple in human physical therapy since the 1970s [1–5]. It is commonly used as an objective measure of joint and muscle disease in addition to being used for patient assessment following joint or muscle trauma [6–8]. Goniometry is also regularly used in the human sector as an objective assessment of healing/improvement in cases of neurologic rehabilitation. Specifically, goniometry has successfully been used in patients receiving rehabilitation for diseases ranging from cerebral palsy [9, 10], to Duchenne Muscular Dystrophy [11], to spinal cord injury [12].

In veterinary medicine, goniometry is used to assess outcome objectively in canine and feline patients undergoing physical therapy while recovering from orthopedic and neurologic disease [13–16]. In the veterinary literature, there remain only a few published reports related to physical rehabilitation and the neurologic patient [17–19]. That being said, neurologic conditions are common in veterinary medicine. One of the most common neurologic diseases for which rehabilitation is a component of therapy is type 1 intervertebral disc disease.

Type 1 intervertebral disc disease was originally described by Hansen in the 1950s [20, 21]. Disc desiccation leads to weakening of the annulus fibrosus and eventual herniation of the nucleus through the annulus and subsequent spinal cord compression. Dachshunds are the most common breed of dog affected by disc disease and herniation [22, 23]. Between 19 and 24% of Dachshunds, within their lifespan, will suffer from thoracolumbar disc disease [22, 23]. The majority of disc herniation occurs in the thoracolumbar spine and results in pelvic limb weakness [24], leading to possible surgery and postoperative rehabilitation therapy. For this reason, there is a significant need for reported range of motion measurements in miniature Dachshunds, as a breed. Having published joint angle measurements for the pelvic limbs in miniature Dachshunds would allow veterinary physical rehabilitation practitioners an objective means to guide and assess physical rehabilitation in their patients. Having objective data on limb girth measurements for miniature Dachshunds presenting with various degrees of pelvic limb paresis would also be prudent as muscle atrophy is a realized sequela to neurologic injury including thoracolumbar disc extrusion.

One difficulty with joint angle measurements in animals versus humans is the variety of limb shape and girth differences among breeds and between animal species. Joint angles differ not only between dogs and cats but also between different dog breeds [13, 14, 25, 26]. To date, the use of goniometry has been validated in both Labrador Retrievers [13] and cats [15]. Joint angles have also been measured and reported for mixed breed dogs and Greyhounds [26]. A study by Benson et al. [25] compared joint angles between two breeds of dog, the Basset hound and the Irish wolfhound. This study determined that there was variability between values for both dog breeds; the final conclusion being that one universal table for normal joint angle values may not be applicable between dog breeds. Thus, there is a need for published range of motion measurements in a variety of dog breeds.

The aims of this paper are (1) to report the mean and median pelvic limb joint angles and limb girth measurements in miniature Dachshunds presenting with ambulatory paraparesis, nonambulatory paraparesis, and paraplegia secondary to thoracolumbar intervertebral disc extrusion and (2) to compare joint angle and limb girth measurements between miniature Dachshunds who present with ambulatory paraparesis, nonambulatory paraparesis, and paraplegia secondary to thoracolumbar intervertebral disc extrusion. It is our hypothesis that we will be able to successfully report the mean and median goniometry and limb girth measurements in miniature Dachshunds with varying degrees of neurologic dysfunction and that there will be no significant difference in these values between groups.

## 2. Materials and Methods

### 2.1. Animals

Dachshunds that presented to the Washington State University Veterinary Teaching Hospital between April 2011 and February 2012 for thoracolumbar disc extrusion were included in the study. Dogs were being recruited for a physical rehabilitation study involving surgically addressed thoracolumbar disc herniation and hydrotherapy. Appropriate client consent was given prior to study enrollment. The project was approved by the Animal Care and Use Committee at the Washington State University. Dogs were excluded from the study if they did not have a thoracolumbar disc extrusion on MR imaging and if they were not taken to surgery.

Dachshunds were split into three groups. Group 1 consisted of 3 dogs; these dogs were ambulatory with pelvic limb paresis at the time of study inclusion. Group 2 consisted of 6 dogs; these dogs were nonambulatory with pelvic limb paresis at the time of study inclusion. Group 3 consisted of 6 dogs; these dogs were paraplegic at the time of study inclusion. All measurements were taken within 24 hours of presentation, diagnostic imaging, and surgery.

### 2.2. Procedures

**Figure 1:**
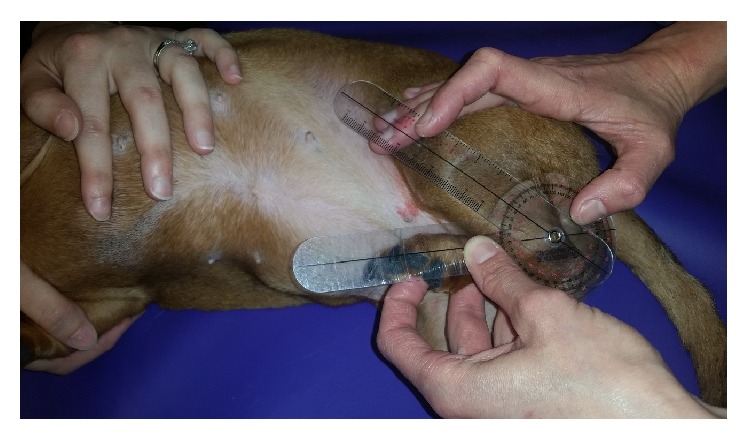


**Figure 2:**
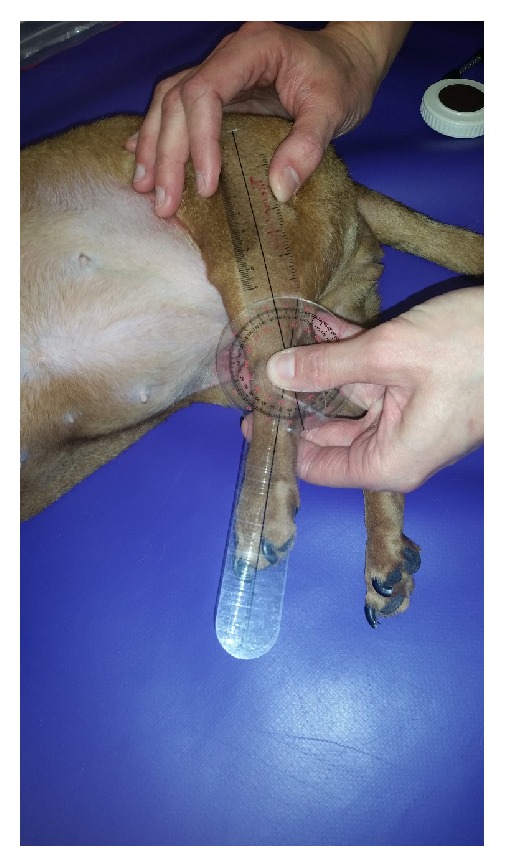


Goniometry was performed using a universal plastic goniometer with 8-inch arms and 360-degree head [14]. Awake dogs were put in lateral recumbency; the angles of extension and flexion on the “up” pelvic limb were measured at the hock, stifle, and hip (see Figures 1 and 2). Angles of flexion and extension were measured a single time. The dog was rotated to the contralateral side and the measurements were repeated on the opposite leg. Joint angle measurements were performed utilizing a previously published and validated technique [13].

**Figure 3:**
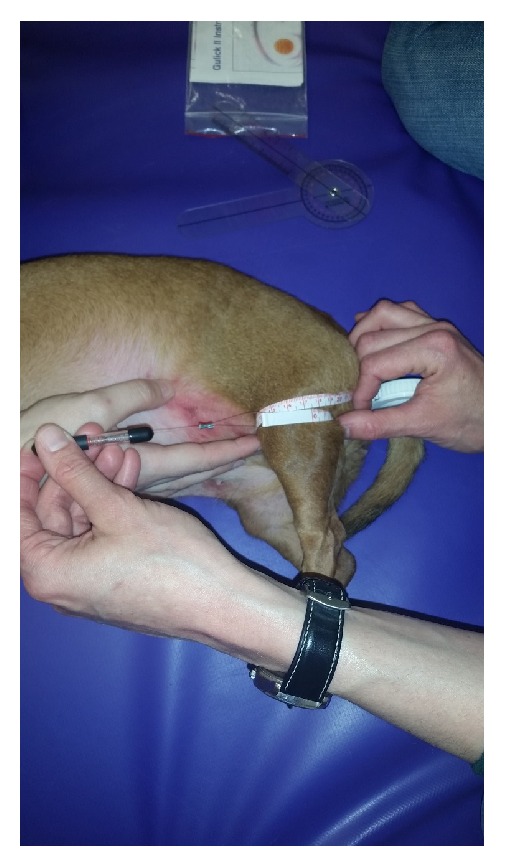


While in lateral recumbency, the girth of both the right and left pelvic limb was also measured in centimeters using a spring tape measure (Gulick II Tape Measure, Fitness Mart, Gays Mills, WI). The technique utilized to perform girth measurements was based on a previously published and validated technique [27]. Thigh length from the greater trochanter to the distal femur at the level of the lateral fabella was measured using the spring tape measure. The thigh circumference was measured 70% distal to the greater trochanter (see Figure 3) [27]. Goniometry and girth measurements were carried out within 24 hours of presentation. Measurements were conducted by one of two certified physical rehabilitation individuals (LAL, SAT), or a resident in neurosurgery, or a board certified neurologist (AVC, SAT).

### 2.3. Statistical Analysis

The statistical analyses were performed using a statistical software package (SAS, version 9.3, SAS Institute, Cary, NC). Mean and standard deviation as well as median values for joint flexion and extension in addition to thigh girth were calculated. Mean and median flexion and extension angles were compared between groups 1–3 for each joint. Each value was viewed independently: dog 1 left limb hock extension was one variable and right limb hock extension was a second variable. The mean and median values for joint flexion and extension in addition to thigh girth were calculated regardless of group. A standard *t*-test was used to assess the data by performing contrasts in the analysis of variance. Each pair of groups 1–3 was compared separately to assess specific differences. *P* values were considered significant at <0.05. Joint angle values were compared for hip, stifle and hock flexion, and extension; girth was also compared. Both goniometry values and girth were compared with a paired *t*-test. A power analysis was done to ensure a sufficient number of dogs were included. The power for this experiment was 0.75 with an alpha of 0.5.

## 3. Results and Discussion

### 3.1. Results

The median and mean angles of flexion at the hip, stifle, and hock for all three groups of dogs are recorded in Table 1. Flexion angles differed between groups to a greater degree when measurements at the hip (median values of 49 to 55 degrees and mean values of 49.1 to 54.3 degrees) and stifle (median values of 43.5 to 54 degrees and mean values of 46 to 56 degrees) were made as compared to measurements at the hock (median values of 39 to 40 degrees and mean values of 39.2 to 39.5 degrees).

The median and mean angles of extension at the hip, stifle, and hock for all three groups of dogs are recorded in Table 2. Similar to flexion angles, extension angles differed between groups to a greater degree when measurements at the hip and stifle were made as compared to the hock. This trend was less obvious for extension as compared to flexion. Median extension values ranged from 151.5 to 160 degrees for the hip and mean values ranged from 152.5 to 156.5 degrees. Median values ranged from 160 to 163.5 degrees for stifle extension, while mean values were 157.3 to 164.2 degrees. Median values for hock extension were 167.5 to 172.5 degrees, while mean values were 167.5 to 171.5 degrees.

The median and mean pelvic limb girth measurements for all three groups of dogs are recorded in Table 3. There was little variation in median and mean limb girth measurements between groups. Median limb girth ranged from 22.5 to 24 cm, while mean girth ranged from 22.7 to 23.7 cm.

There were no significant differences in joint angles or girth among the three groups (ambulatory paraparetic, nonambulatory paraparetic, or paraplegic); however, statistical significance was not reached; *P* values ranged from 0.27 to 0.99. When group was disregarded and values for extension, flexion, and girth combined, no differences existed.

### 3.2. Discussion

We were able to successfully measure joint angles and girth in miniature Dachshunds in this study. It was found that when angles of flexion and extension between the three groups of dogs were compared, there was a trend toward measurements being more similar when made at the hock versus the stifle or hip. In our study, a variety of individuals made joint angle measurements, the trend in the data would support the idea of less variability when measurements of flexion and extension at the hock are made as compared to measurements at the stifle or hip in miniature Dachshunds. This finding is different than previous canine goniometry studies wherein measurements at all joints were found to be repeatable between individuals [13]. Jaegger et al. [13] found there to be high intraobserver agreement between staff members when goniometry was performed in Labrador Retrievers. One explanation for this difference in miniature Dachshunds could be the shape of the pelvic limb in this breed and its associated muscle mass. In this chondrodystrophoid breed, the bony landmarks utilized to make reliable goniometry measurements are more challenging to palpate and reliably locate as compared to the same structures in a long-legged breed, such as the Labrador Retriever. Similarly, the location of the thick pelvic limb thigh musculature in relationship to the inguinal fold, inherent to the miniature Dachshund pelvic limb, made acquisition of limb girth measurements challenging.

It is unlikely that the trend toward increased range of joint angles measured in the hip and stifle as compared to the hock between groups was secondary to the neurologic status of the patients. There was no trend toward one group of dogs having greater or lesser joint angles at one joint as compared to the next.

Additionally, there was no significant degree of variability in limb girth between groups of miniature Dachshunds enrolled in our study. Also, no trend was observed with respect to neurologic status affecting limb girth. Patients in this study suffered from thoracolumbar disc herniation and presented acutely after injury for work up and surgery. Thus, it is unlikely either disuse or neurogenic atrophy, both of which would affect limb girth measurements, was contributing to limb girth values in these dogs.

Limb girth measurements proved to be challenging in this population of dogs. In this study, thigh circumference was measured at a location 70% distal to the greater trochanter [27]. A previous study looking at circumference as measured at two separate locations along the femur (50% versus 70% of femur length) showed it to be technically easier to make reliable measurements at a distance 70% of femur length. The researchers postulated that because this more distal location is farther from the skin of the flank it is easier to get reliable measurements [28]. The unique shape of the Dachshund leg and the close proximity of flank skin to the stifle made measurement of girth challenging.

## 4. Conclusion

Some major goals of rehabilitation in neurologic patients are maintenance of muscle strength and joint mobility in addition to the reduction of muscle atrophy. Objective data such as joint angle and limb girth measurements is vital to gaging rehabilitation success in miniature Dachshunds suffering from both orthopedic and neurologic injuries. We conclude that joint angle and limb girth measurements can successfully be made in the miniature Dachshund but that the unique shape and muscle distribution of the breed's pelvis make obtaining these values challenging. We also conclude that miniature Dachshunds, with varying neurologic dysfunction at the time of range of motion and limb girth assessments, show no significant difference in values when measurements are made within 24 hours of acute onset of neurologic signs.

## Figures and Tables

**Table 1:** Median and mean (with standard deviation) flexion angles at the hip, stifle, and hock in miniature Dachshunds. Each value was viewed independently, meaning dog 1 left limb hock flexion was one variable and right limb hock flexion was a second variable.

Group	Median flexion hip (°)	Median flexion stifle (°)	Median flexion hock (°)	Mean flexion hip (°)	Standard deviation	Mean flexion stifle (°)	Standard deviation	Mean flexion hock (°)	Standard deviation
Group 1: ambulatory paraparetic group	55	54	39	54.3	4.6	56	12.9	39.2	5.3
Group 2: nonambulatory paraparetic group	49	50	40	49.1	8.4	48.2	13.2	39.5	7.1
Group 3: paraplegic group	50	43.5	40	51.3	11.9	46	11.0	39.3	5.7
Pooled data combining all three groups	50	50	40	51.6	2.61	50.1	5.3	39.3	0.15

**Table 2:** Median and mean (with standard deviation) extension angles at the hip, stifle, and hock in miniature Dachshunds. Each value was viewed independently, meaning dog 1 left limb hock extension was one variable and right limb hock extension was a second variable.

Group	Median extension hip (°)	Median extension stifle (°)	Median extension hock (°)	Mean extension hip (°)	Standard deviation	Mean extension stifle (°)	Standard deviation	Mean extension hock (°)	Standard deviation
Group 1: ambulatory paraparetic group	155	163.5	167.5	155	9.4	164.2	6.9	169.2	9.2
Group 2: nonambulatory paraparetic group	151.5	160	172.5	152.5	10.6	157.3	7.7	171.5	8.0
Group 3: paraplegic group	160	160	167.5	156.5	10.1	159.4	10.0	167.5	9.7
Pooled data combining all three groups	155	160	167.5	154.7	2.0	160.3	3.5	169.4	2.0

**Table 3:** Median and mean (with standard deviation) thigh limb girth measurements in miniature Dachshunds. All limb girth measurements were made along the femur at a location 70% distal to the greater trochanter. Each value was viewed independently, meaning dog 1 left limb girth was one variable and right limb girth was a second variable.

Group	Median limb girth (cm)	Mean limb girth (cm)	Standard deviation
Group 1: ambulatory paraparetic group	23.5	22.8	4.3
Group 2: nonambulatory paraparetic group	22.5	22.7	2.2
Group 3: paraplegic group	24	23.7	3.7
Pooled data combining all three groups	23.5	23.1	0.55

## Abbreviation List

WSU-VTH: Washington State University Veterinary Teaching Hospital.
